# Non-invasive islet β-cell markers track with weight-loss interventions for type 2 diabetes: a prospective cohort study

**DOI:** 10.1038/s41366-026-02070-x

**Published:** 2026-04-08

**Authors:** Hongyan Sun, Yun Shen, Susan J. Burke, Phillip Brantley, Ricky Brock, Dachuan Zhang, Shengping Yang, Gang Hu, J. Jason Collier

**Affiliations:** Pennington Biomedical Research Center, Baton Rouge, LA USA

**Keywords:** Obesity, Type 2 diabetes

## Abstract

**Objective:**

This study investigated the longitudinal impact of intensive medical intervention (IMI) and bariatric surgery procedures on indirect measures of pancreatic β-cell death and function.

**Methods:**

Eighty-four participants (28 non-type 2 diabetes [T2D], 56 T2D) from the HEADS UP study were assessed at baseline and 1-year post-intervention. Circulating unmethylated and methylated insulin gene [*INS*] DNA were quantified from blood samples via droplet digital polymerase chain reaction (PCR). Metabolic biomarkers, including fasting plasma glucose, HbA1c, proinsulin-to-insulin ratio, insulin, and C-peptide, were analyzed.

**Results:**

At baseline, participants with T2D had significantly higher levels of unmethylated *INS* DNA and higher unmethylated-to-methylated *INS* DNA ratios than individuals without T2D. After 1-year, significant reductions in these biomarkers were observed primarily in the T2D group. Bariatric surgeries yielded greater improvements in metabolic profiles and reductions in unmethylated *INS* DNA than IMI. Despite substantial metabolic improvement, participants with T2D maintained elevated proinsulin-to-insulin ratios, indicating alterations to β-cell function.

**Conclusions:**

Circulating unmethylated *INS* DNA is a non-invasive index of β-cell death and responds to weight-loss interventions. Metabolic surgeries are more effective than IMI in preserving β-cell mass and function, highlighting their potential in diabetes management. Long-term studies are necessary to confirm these initial findings.

## Introduction

Type 2 diabetes (T2D) is characterized by progressive insulin resistance and insufficient pancreatic β-cell function, leading to significant global morbidity and mortality [1]. Early detection and monitoring of islet β-cell dysfunction remain critical for improving clinical outcomes. Pancreatic β-cell insulin secretion in response to blood glucose fluctuations maintains glucose homeostasis, while β-cell mass represents the total functional β-cell population influenced by proliferation, differentiation, and cell death. Recently, circulating unmethylated insulin gene (*INS*) DNA has emerged as a promising biomarker for pancreatic β-cell death in Type 1 diabetes (T1D) [2, 3], but has not been examined in study participants with T2D.

Metabolic surgery, including vertical sleeve gastrectomy (VSG) and Roux-en-Y gastric bypass (RYGB), is a key therapeutic strategy for generating substantial weight loss and improved glycemic control with islet β-cell preservation [4]. The Diabetes Remission Clinical Trial (DiRECT) [5] reported that diet-induced weight loss resulted in remission of T2D in nearly half of the participants by restoring β-cell function and peripheral insulin sensitivity. The STAMPEDE trial [6–8] demonstrated superior β-cell recovery following surgical procedures compared to medical therapy alone, with improvements persisting beyond 5 years. Collectively, the differential impacts of separate weight-loss approaches compared with intensive medical interventions (IMI) on circulating markers of β-cell loss remain inadequately characterized. Thus, this study aimed to compare changes in unmethylated and methylated *INS* DNA in serum and evaluate associated endocrine, anthropometric, and metabolic parameters across participants with and without T2D and with obesity undergoing IMI, VSG, or RYGB interventions.

## Materials and methods

### Study subjects and samples

Participants for the current analysis were drawn from the HEADS UP study, a prospective obesity management trial conducted from July 2011 to June 2016 and supported by the State of Louisiana [9]. The HEADS UP study aimed primarily to assess and compare the health outcomes of surgical versus intensive medical strategies for severe obesity among adult beneficiaries of the Louisiana Office of Group Benefits (OGB). The detailed protocol, including comprehensive descriptions of study design, participant recruitment, and methodological approaches, has been published previously [9–11]. Briefly, eligibility for participation required individuals to be adults aged 20–64 years with severe obesity, defined by specific body mass index (BMI) thresholds: BMI ≥ 40 kg/m² without T2D or BMI ≥ 35 kg/m² accompanied by T2D for the surgical intervention groups, and BMI ≥ 33 kg/m² for those participating in the IMI program. Participants were excluded based on the following criteria: incomplete screening procedures (web-based or telephone follow-ups), failure to meet the necessary BMI eligibility requirements, inability to demonstrate a preliminary weight reduction of at least 4 pounds during pre-screening, pregnancy or planned pregnancy within 3 years of the study initiation, significant underlying medical conditions or psychiatric illnesses, or prior history of bariatric surgery. They were not excluded once they successfully completed screening.

The current analysis comprised 84 participants, including 28 without T2D and 56 with T2D. Inclusion criteria for this subgroup analysis included complete fasting biospecimens obtained at baseline and at the 1-year follow-up, along with comprehensive clinical data recorded at both baseline and 1 year. All enrolled participants provided written informed consent. Ethical approval of the study protocol was granted by the Institutional Review Board (IRB) at Pennington Biomedical Research Center and all methods were performed according to institutional and IRB guidelines.

### Anthropometric and lab measures

Demographic variables, including age, gender, and race, along with comprehensive clinical measurements such as body weight, waist circumference, blood pressure, fasting plasma glucose, glycated hemoglobin (HbA1c), and cholesterol levels, were obtained at baseline and again at the 1-year follow-up visits. All anthropometric measurements, including height, body weight, and waist circumference, were performed by trained study personnel using standardized protocols. Specifically, participants were instructed to wear lightweight clothing and remove footwear during height and weight assessments. Body mass index (BMI) was subsequently calculated as weight (in kilograms) divided by height squared (in meters). Blood pressure readings were taken according to established guidelines and standardized procedures. Homeostatic Model Assessment for Insulin Resistance (HOMA-IR) was calculated using the equation: fasting glucose (mmol/L) × fasting insulin (mU/L) divided by 22.5 [12, 13]. Homeostatic Model Assessment of beta-cell function (HOMA-β) was determined by the equation: (20 × fasting insulin [mU/L]) divided by (fasting glucose [mmol/L] – 3.5). The proinsulin-to-insulin ratio was calculated after converting the unit of insulin from mU/L to pmol/L. All participants who underwent surgical interventions ceased their diabetes medications immediately upon surgery. In contrast, participants in the IMI group typically either discontinued their diabetes medications or significantly reduced their doses post-intervention, with metformin being the most commonly maintained medication at lower dosages following weight loss. Fasting blood samples for biochemical analyses were collected from all participants at baseline and at the 1-year follow-up appointment. Diabetes status was determined by self-reported diagnosis and was further confirmed according to the American Diabetes Association guidelines [14], defined by fasting plasma glucose ≥7.0 mmol/L, HbA1c ≥ 6.5%, or active treatment with diabetes medications.

### DNA isolation and bisulfite conversion

Cell-free DNA was isolated from 200 μl serum samples using QIAamp DNA Blood Mini kit (Qiagen, Cat# 51106). All procedures followed the kit protocol, and DNA was eluted using 60 μl of elution buffer. A total of 20 μl of the isolated DNA underwent bisulfide conversion using the EZ DNA Methylation-Lightning Kit (ZYMO, Cat# D5030), following the manufacturer’s instructions.

### Droplet digital PCR (ddPCR)

The Life Technologies Applied Biosystem SNP Taqman primer/probe mix for *INS* DNA (Thermo Fisher Scientific, Cat# 4332075), ddPCR supermix (Bio-Rad, Cat# 1863010), EcoRI, and 2.5 μl of bisulfite-converted DNA were assembled for multiplex ddPCR. The FAM probe and VIC probe were used to detect unmethylated and methylated DNA, respectively. Droplets were generated using a Bio-Rad oil droplet generator and then transferred to a 96-well plate for PCR. The PCR products were read using the QX200 Droplet Reader and analyzed with QuantaSoft software (Bio-Rad). For each sample, the concentrations of unmethylated and methylated DNA were collected for further analysis.

### Human proinsulin, insulin and C-peptide measurements

Serum insulin (Cat # 10-1113-01), proinsulin (Cat # 10-1118-01), and C-peptide (Cat # 10-1136-01) concentrations were quantified using commercially available enzyme-linked immunosorbent assay (ELISA) kits provided by Mercodia, strictly adhering to the manufacturer’s guidelines. Samples were incubated with enzyme-conjugated detection antibodies specific to insulin, proinsulin, or C-peptide, followed by a substrate reaction producing a measurable colorimetric signal. The intensity of this signal, directly proportional to analyte concentration, was then quantified using a Glomax spectrophotometric plate reader (Promega) at the appropriate absorbance wavelength, enabling accurate and sensitive biomarker measurements.

### Statistical analysis

Continuous variables were summarized using means ± standard deviations (SD), and categorical variables were presented as numbers (percentages). To evaluate the differences in baseline characteristics between participants with and without T2D, Student’s *t* test was used for normally distributed continuous variables, and the Mann–Whitney *U* test was applied for variables with non-normal distributions. Chi-square tests or Fisher’s exact tests were employed for categorical variables as appropriate. To assess changes in metabolic and β-cell-related parameters, including weight, BMI, waist circumference, systolic blood pressure, fasting plasma glucose, HbA1c, HOMA-IR, HOMA-β, proinsulin-to-insulin ratio, and levels of unmethylated and methylated *INS* DNA from baseline to 1-year follow-up, paired *t*-tests or Wilcoxon signed-rank tests were performed within each intervention group, depending on the normality of data distribution. Comparisons among the three intervention groups (IMI, VSG, and RYGB) were conducted using analysis of variance (ANOVA) with Bonferroni corrections for pairwise comparisons or Kruskal–Wallis tests, followed by Dunn’s post hoc analyses for non-normally distributed data. Pearson correlation analysis was performed to evaluate the linear associations between baseline weight and baseline levels of proinsulin, insulin, unmethylated *INS* DNA, and methylated *INS* DNA, as well as between the changes in weight and changes in proinsulin and insulin levels. Data were visually examined for linearity, and Pearson correlation coefficients (*r*) were calculated to quantify the strength and direction of these associations. Statistical significance was defined as a two-tailed *p* value < 0.05. All statistical analyses were conducted using R statistical software (version 4.2.0).

## Results

### Characteristics of the baseline study participants

Two groups of participants were included in this study: 28 without T2D and 56 with T2D (Supplementary Fig. 1). Baseline characteristics of the study participants, including age, sex, race, weight, BMI, blood pressure, weight-loss procedures, fasting plasma glucose, HbA1c, total cholesterol, triglycerides, low-density lipoprotein (LDL) cholesterol, fasting insulin, fasting proinsulin, proinsulin/insulin ratio, fasting C-peptide, HOMA-IR, and HOMA-β, were described and compared in Supplementary Table 1. Compared to participants without T2D, fasting plasma glucose and HbA1c levels were significantly higher in those with T2D, indicating clinical parameters associated with established disease. This is consistent with previous studies [15].

### Analysis of unmethylated INS DNA in participants with and without T2D between baseline and 1-year

**Fig. 1:**
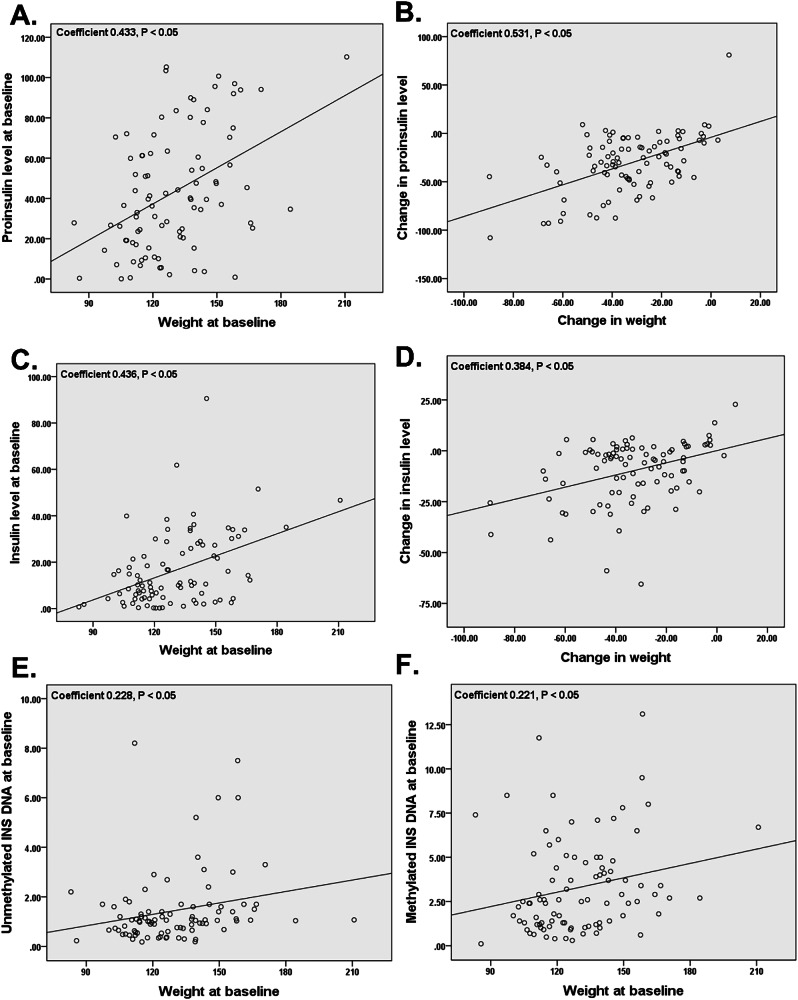
Circulating insulin and measurements of *INS* DNA correlate with body weight. **A** Proinsulin (pmol/L) expressed as a function of weight at baseline. **B** Change in proinsulin levels plotted against changes in weight. **C** Insulin expressed as a function of weight at baseline. **D** Change in circulating insulin plotted against changes in weight. **E**, **F** Correlation between baseline unmethylated or methylated insulin DNA and weight at baseline. *p* < 0.05 was considered statistically significant.

The presence of cell-free DNA in blood has been valuable for understanding changes occurring in vivo during various interventions and disease states [16, 17]. We measured methylated and unmethylated DNA regions using droplet digital PCR (ddPCR) corresponding to the insulin gene as a proxy for changes in islet β-cell number. At baseline, unmethylated *INS* DNA levels were significantly higher in participants with T2D compared to those without T2D (Supplementary Table 1). Similarly, the ratio of unmethylated to methylated *INS* DNA was significantly elevated in participants with T2D (Supplementary Table 1). We also analyzed changes in metabolic profiles, including weight, BMI, waist circumference, systolic blood pressure, fasting plasma glucose, HbA1c, HOMA-IR, and HOMA-β, between baseline and 1 year (Table 1). Using Pearson correlations, we found that proinsulin was significantly correlated with both weight at baseline (Fig. 1A) and change in body weight (Fig. 1B). This was also true for mature processed insulin (Fig. 1C, D). We discovered that both unmethylated (Fig. 1E) and methylated (Fig. 1F) DNA regions corresponding to the insulin gene were modestly correlated with weight at baseline. In individuals with T2D, unmethylated *INS* DNA levels significantly decreased at 1 year compared to baseline (Table 1). Furthermore, the ratio of unmethylated to methylated *INS* DNA was significantly lower at 1 year compared to baseline in the T2D group (Table 1).

**Table 1:** Changes from baseline to Year 1 in metabolic profiles and serum parameters.

	Total	Non-diabetes	Type 2 diabetes
Weight, kg	−33.1 ± 19.3	−33.0 ± 24.0	−33.1 ± 17.1
Body mass index, kg/m^2^	−11.8 ± 6.53	−11.8 ± 7.92	−11.8 ± 5.88
Waist circumference, cm	−24.3 ± 14.7	−24.6 ± 18.8	−24.1 ± 12.7
Systolic blood pressure, mmHg	−9.23 ± 16.9	−10.6 ± 15.8	−8.6 ± 17.5
Fasting plasma glucose, mmol/L	−1.23 ± 1.88	−0.77 ± 0.82	−1.43 ± 2.17
Hemoglobin A1c, %	−0.9 ± 1.1	−0.5 ± 0.3	−1.0 ± 1.2
HOMA-IR	−0.17 ± 0.24	−0.2 ± 0.27	−0.16 ± 0.23
HOMA-β	−2.58 ± 7.67	−3.46 ± 8.89	−2.19 ± 7.06
Fasting insulin, mU/L	−9.80 ± 15.1	−13.2 ± 19.3	−8.26 ± 12.7
Fasting proinsulin, pmol/L	−31.1 ± 29.9	−32.6 ± 37.4	−30.4 ± 26.1
Proinsulin/insulin	−1.73 ± 33.5	−8.64 ± 26.6	1.38 ± 35.9
Unmethylated *INS* DNA, copies/μL	−0.32 ± 1.11	0.04 ± 0.77	−0.49 ± 1.20*
Methylated *INS* DNA, copies/μL	−0.59 ± 2.70	−0.34 ± 2.48	−0.70 ± 2.80
Unmethylated/Methylated *INS* DNA ratio	−0.04 ± 0.41	0.02 ± 0.39	−0.07 ± 0.41*

All data were mean ± SD.

*INS DNA* insulin gene DNA, *HOMA-IR* homeostasis model assessment-estimated insulin resistance, *HOMA-β* homeostatic model assessment of β-cell function.

*Non-diabetes group vs. Type 2 diabetes group, *p* < 0.05.

### Assessment of unmethylated and methylated INS DNA in different weight loss procedures

The metabolic profiles and serum parameters were analyzed between baseline and 1-year follow-ups for participants undergoing three weight-loss interventions: IMI, VSG, and RYGB (Table 2). The average weight loss was −16.1 ± 13.8 kg for IMI, −34.4 ± 12.8 kg for VSG, and −48.7 ± 15.6 kg for RYGB. Changes in BMI, waist circumference, systolic blood pressure, fasting plasma glucose, HbA1c, HOMA-IR, and HOMA-β between baseline and 1 year for each group are detailed in Table 2. Additionally, serum levels of proinsulin-to-insulin ratios, unmethylated *INS* DNA, and methylated *INS* DNA were measured. Both unmethylated *INS* DNA and the ratio of unmethylated to methylated *INS* DNA had greater decreases from baseline to 1-year follow-up in the bariatric surgery groups compared to the IMI group.

**Table 2:** Changes from baseline to Year 1 in metabolic profiles and serum parameters by different weight loss procedures.

	Intensive medical intervention	Sleeve gastrectomy	Roux-en-Y gastric bypass
Total population, *N* = 84			
Weight, kg	−16.1 ± 13.8	−34.4 ± 12.8	−48.7 ± 15.6
Body mass index, kg/m^2^	−5.58 ± 4.63	−12.6 ± 4.03	−17.2 ± 4.67
Waist circumference, cm	−11.4 ± 11.4	−25.6 ± 10.0	−35.6 ± 11.6
Systolic blood pressure, mmHg	−3.30 ± 18.4	−11.7 ± 15.3	−12.6 ± 15.7
Fasting plasma glucose, mmol/L	−0.99 ± 1.77	−1.53 ± 1.87	−1.15 ± 2.00
Hemoglobin A1c, %	−0.6 ± 0.9	−1.0 ± 1.2	−1.0 ± 1.1
HOMA-IR	−0.09 ± 0.21	−0.22 ± 0.25	−0.21 ± 0.24
HOMA-β	−0.17 ± 7.89	−4.99 ± 8.50	−2.94 ± 5.61
Fasting insulin, mU/L	−4.15 ± 12.6	−12.7 ± 15.5	−12.4 ± 15.8
Fasting proinsulin, pmol/L	−19.7 ± 29.7	−32.9 ± 25.2	−40.5 ± 31.4
Proinsulin/insulin	−4.96 ± 15.7	6.37 ± 50.7	−6.61 ± 22.9
Unmethylated *INS* DNA, copies/μL	−0.22 ± 0.77	−0.36 ± 1.29	−0.39 ± 1.24
Methylated *INS* DNA, copies/μL	−0.47 ± 2.52	−0.56 ± 2.57	−0.73 ± 3.06
Unmethylated/Methylated *INS* DNA ratio	0.02 ± 0.35	−0.01 ± 0.36	−0.14 ± 0.48
Participants without diabetes, *N* = 28			
Weight, kg	−13.7 ± 13.4	−36.6 ± 6.74	−58.3 ± 22.8
Body mass index, kg/m^2^	−4.81 ± 4.51	−14.2 ± 2.77	−19.9 ± 6.03
Waist circumference, cm	−8.46 ± 10.6	−27.8 ± 5.15	−45.5 ± 14.6
Systolic blood pressure, mmHg	−4.8 ± 15.0	−12.3 ± 14.6	−17.3 ± 16.8
Fasting plasma glucose, mmol/L	−0.67 ± 0.54	−1.25 ± 1.12	−0.45 ± 0.71
Hemoglobin A1c, %	−0.5 ± 0.2	−0.6 ± 0.4	−0.5 ± 0.3
HOMA-IR	−0.04 ± 0.14	−0.37 ± 0.28	−0.28 ± 0.32
HOMA-β	−1.37 ± 6.72	−10.8 ± 10.7	−3.29 ± 4.21
Fasting insulin, mU/L	−1.90 ± 11.0	−22.9 ± 17.9	−20.4 ± 23.1
Fasting proinsulin, pmol/L	−6.05 ± 31.2	−43.6 ± 21.5	−61.2 ± 33.2
Proinsulin/insulin	−7.00 ± 22.2	−2.33 ± 5.35	−17.3 ± 42.3
Unmethylated *INS* DNA, copies/μL	−0.21 ± 0.63	0.37 ± 1.01	0.10 ± 0.63
Methylated *INS* DNA, copies/μL	−0.49 ± 2.98	−0.26 ± 1.49	−0.21 ± 2.75
Unmethylated/Methylated *INS* DNA ratio	−0.06 ± 0.46	0.13 ± 0.42	0.02 ± 0.22
Participants with diabetes, *N* = 56			
Weight, kg	−17.7 ± 14.3	−33.6 ± 14.5	−45.2 ± 10.8
Body mass index, kg/m^2^	−6.10 ± 4.77	−12.1 ± 4.32	−16.2 ± 3.78
Waist circumference, cm	−13.5 ± 11.8	−24.8 ± 11.3	−32.0 ± 8.02
Systolic blood pressure, mmHg	−2.3 ± 20.7	−11.5 ± 15.9	−11.0 ± 15.4
Fasting plasma glucose, mmol/L	−1.22 ± 2.24	−1.64 ± 2.09	−1.41 ± 2.26
Hemoglobin A1c, %	−0.7 ± 1.2	−1.2 ± 1.3	−1.1 ± 1.2
HOMA-IR	−0.12 ± 0.25	−0.17 ± 0.22	−0.19 ± 0.22
HOMA-β	−0.63 ± 8.72	−2.85 ± 6.61	−2.81 ± 6.11
Fasting insulin, mU/L	−5.65 ± 13.7	−9.09 ± 13.1	−9.56 ± 11.6
Fasting proinsulin, pmol/L	−28.8 ± 25.5	−29.1 ± 25.8	−32.9 ± 27.8
Proinsulin/insulin	−3.61 ± 9.81	9.54 ± 59.1	−2.69 ± 8.33
Unmethylated *INS* DNA, copies/μL	−0.56 ± 1.37	−0.62 ± 1.30	−0.23 ± 0.86
Methylated *INS* DNA, copies/μL	−0.93 ± 3.20	−0.67 ± 2.89	−0.46 ± 2.25
Unmethylated/Methylated *INS* DNA ratio	0.01 ± 0.39	−0.05 ± 0.34	−0.18 ± 0.50

All data were mean ± SD.

*INS DNA* insulin gene DNA, *HOMA-IR* homeostasis model assessment-estimated insulin resistance, *HOMA-β* homeostatic model assessment of β-cell function.

In participants without T2D, the average weight loss was −13.7 ± 13.4 kg (IMI), −36.6 ± 6.74 kg (VSG), and −58.3 ± 22.8 kg (RYGB). For those with T2D, the average weight loss was −17.7 ± 14.3 kg (IMI), −33.6 ± 14.5 kg (VSG), and −45.2 ± 10.8 kg (RYGB). Changes in metabolic profiles for participants with and without T2D are presented separately in Table 2. The ratio of proinsulin to insulin was elevated across all three weight-loss interventions when comparing participants with T2D to those without T2D (Table 2). In people without T2D, both unmethylated *INS* DNA levels and the ratio of unmethylated to methylated *INS* DNA had greater decreases from baseline to 1-year follow-up in the bariatric surgery groups compared to the IMI group.

**Fig. 2:**
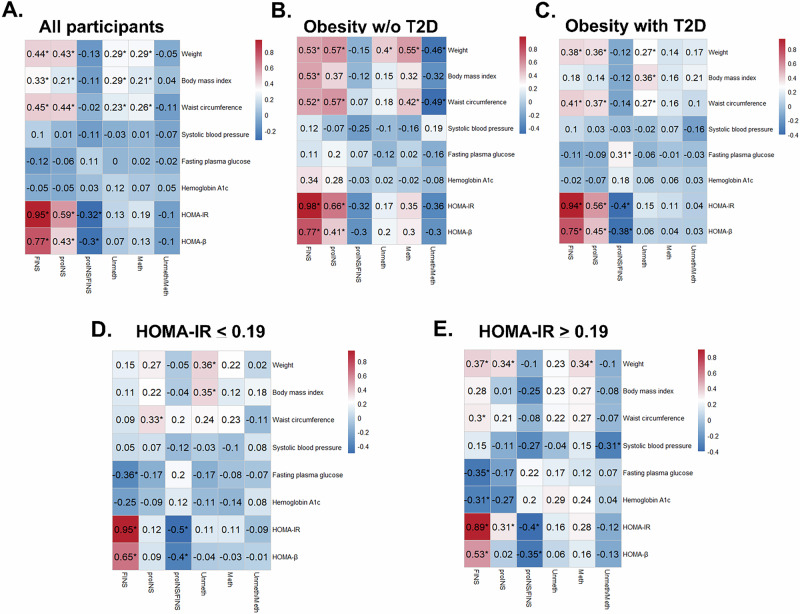
Correlation heatmaps of clinical characteristics among participants across different subgroups. **A** All participants; **B** Individuals with obesity without type 2 diabetes (T2D); **C** Individuals with obesity and T2D; **D** Participants with lower insulin resistance (HOMA-IR < 0.19); and **E** Participants with higher insulin resistance (HOMA-IR ≥ 0.19). *, *p* < 0.05.

We next evaluated how well HOMA correlated with several discrete metabolic variables (Fig. 2 and Supplementary Fig. 2). This included the use of both HOMA-IR and HOMA-β [12]. Such analyses revealed that BMI and waist circumference significantly correlated with both proinsulin and fasting insulin (Fig. 2A). In addition, when all study participants were considered together, HOMA-IR and HOMA-β were positively correlated with proinsulin and fasting insulin (Fig. 2A). These findings held true for surgical weight loss interventions (Supplementary Fig. 2).

We next separated study participants by obesity without (Fig. 2B) or with accompanying T2D (Fig. 2C). In this analysis, there was also a significant correlation between HOMA-IR with both fasting insulin and proinsulin. This relationship also held when comparing HOMA-β with fasting insulin and proinsulin (Fig. 2B, C). When the data were stratified using HOMA-IR values less than 0.19 versus values greater than 0.19 , we observed a significant correlation between HOMA-IR and proinsulin only in the group with HOMA-IR values greater than 0.19 (Fig. 2D). Fasting insulin was significantly correlated with HOMA-IR and HOMA-β in both groups (Fig. 2D, E). In addition, proinsulin and fasting insulin significantly correlate with body weight only in the group with HOMA-IR greater than 0.19 (Fig. 2D).

## Discussion

Changes in pancreatic islet β-cell mass and function are key variables controlling T2D progression and reversal during weight loss [18]. This study demonstrated that circulating unmethylated *INS* DNA, a marker for β-cell loss, is significantly elevated at baseline in participants with T2D compared to those without the disease, indicating it is a sensitive marker of pancreatic β-cell loss. Following 1 year of weight loss intervention, patients with T2D experienced significant reductions in serum unmethylated *INS* DNA levels and the ratio of unmethylated to methylated *INS* DNA. These biomarkers correlate with total body mass (Fig. 1), underscoring the potential of targeted weight-loss interventions to alleviate islet β-cell damage in specific populations, including those with T2D. Importantly, participants undergoing bariatric surgical procedures (VSG and RYGB) exhibited greater improvements in metabolic profiles and more pronounced reductions in circulating unmethylated *INS* DNA compared to IMI alone, suggesting greater weight loss drives improved islet β-cell function and reduces β-cell death.

In a porcine model, RYGB lead to significant improvements in glycemic control, attributed to increases in β-cell mass, islet number, and extra islet β-cells [19]. Clinical trials have demonstrated that RYGB improves β-cell function more effectively than intensive lifestyle interventions, with enhanced insulin secretion and processing, occurring independently of weight loss [20, 21]. The current study is the first population-based data set reporting improvements in circulating β-cell death markers alongside circulating indices of islet β-cell function using different weight loss interventions.

Our findings align with previous evidence indicating that elevated proinsulin is an indicator of insulin resistance and harbinger of T2D [22]. In addition, the present data emphasize the metabolic benefits of bariatric surgery as a weight loss intervention supporting T2D remission, with clear improvements in metabolic health and cellular markers of pancreatic β-cell function. Our data also agree with reports showing that drastic reductions in caloric intake improve islet β-cell function [23]. Indeed, the restoration of islet β-cell function in T2D patients after weight loss emphasizes the ongoing need to enhance therapeutic strategies by combining substantial weight loss, pharmacological interventions, and lifestyle modifications. Such interventions will likely be patient-specific, which will require future precision medicine strategies.

Importantly, our data highlighted the differential impact of distinct interventions between groups with and without T2D during obesity. Study participants with T2D demonstrated persistently elevated proinsulin-to-insulin ratios across interventions, indicating relatively slower recovery time for β-cell function relative to metabolic improvements. These findings highlight the complexity of T2D pathology, wherein islet β-cell recovery may require additional steps beyond just weight loss (e.g., decreases in pancreatic fat, improvements in peripheral insulin sensitivity, etc.) for complete restoration of physiological insulin secretion.

One such mechanism for restoring β-cell function is the alteration of gut hormone profiles, particularly the increased secretion of glucagon-like peptide-1 (GLP-1) and peptide YY (PYY) [24]. GLP-1 signaling in the pancreas enhances insulin secretion, promotes β-cell proliferation, and inhibits apoptosis, thereby improving overall β-cell function and mass. GLP-1 agonists also promote weight loss through pharmacologic means [25], providing additional avenues complementary to surgical and lifestyle interventions. Importantly, reducing caloric intake is also expected to alleviate nutritional stressors on islet β-cells, allowing for recovery of secretory function over time. Thus, weight loss through distinct mechanisms, including metabolic surgery, GLP-1 agonists, and very low-calorie diets alleviate tissue lipid burden, chronic inflammation and oxidative stress, all of which are detrimental to β-cell health [26]. Together, we view such mechanisms as contributors to the observed improvements in β-cell biomarkers and metabolic parameters following weight loss.

Our study has several strengths, including a longitudinal follow-up design and robust biomarker analyses, but also bears notable limitations. Primarily, the study duration of 1 year may be insufficient to capture the full trajectory of β-cell recovery. Over this 1 year, we only have baseline and the outcomes at the 1-year mark, rather than continuous measurements during the full year. Additionally, participant heterogeneity regarding diabetes duration, medication use, and adherence could have influenced the observed biomarker levels and metabolic outcomes.

In conclusion, circulating unmethylated *INS* DNA represents a valuable non-invasive biomarker, responsive to weight-loss interventions, by which to indirectly assess changes in islet β-cell death. Our study shows that ddPCR measurements in conjunction with measures associated with islet β-cell function, such as fasting insulin and proinsulin, provide discrete and complementary non-invasive outcomes relevant to understanding the pathophysiology of T2D and the impact of weight loss on disease reversal.

## Supplementary information

supplementary material

## Data Availability

The datasets generated and/or analyzed during the current study are available from the corresponding author on reasonable request.
