# Supplementary material for: Non-invasive islet β-cell markers track with weight-loss interventions for type 2 diabetes: a prospective cohort study
Source: Int J Obes (Lond). 2026 Apr 8;50(6):1322–8. doi: 10.1038/s41366-026-02070-x (PMC13287002; doi:10.1038/s41366-026-02070-x)
Supplement: Supplementary file 1 — supplementary material [file 41366_2026_2070_MOESM1_ESM.docx]

**Non-invasive islet β-cell markers track with weight-loss interventions for type 2 diabetes: a prospective cohort study**

Hongyan Sun^1^, Yun Shen^1^, Susan J. Burke^1^, Phillip Brantley^1^, Ricky Brock^1^, Dachuan Zhang^1^, Shengping Yang^1^, Gang Hu^1*^, and J. Jason Collier^1*^

^1^Pennington Biomedical Research Center, Baton Rouge, Louisiana, USA

**Supplementary Table 1**. Baseline characteristics of the study participants.

|  | Total  N=84 | Non-diabetes  N=28 | Type 2 diabetes  N=56 |
| --- | --- | --- | --- |
| Age, yr | 50.7±8.46 | 49.9±8.15 | 51.1±8.63 |
| Sex, n (%) |  |  |  |
| Men | 20 (22.2) | 6 (21.4) | 12 (21.4) |
| Women | 70 (77.8) | 22 (78.6) | 44 (78.6) |
| Race, n (%) |  |  |  |
| White | 67 (79.8) | 23 (82.1) | 44 (78.6) |
| African American | 17 (20.2) | 5 (17.9) | 12 (21.4) |
| Weight, kg | 130±21.7 | 129±25.2 | 130±20.1 |
| Body mass index, kg/m^2^ | 46.8±5.85 | 47.3±6.20 | 46.6±5.72 |
| Waist circumference, cm | 133±14.6 | 133±16.8 | 132±13.4 |
| Blood pressure, mmHg |  |  |  |
| Systolic | 127±14.0 | 127±11.7 | 127±15.1 |
| Diastolic | 81.0±9.37 | 82±8.0 | 80±10.0 |
| Weight loss procedures, n (%) |  |  |  |
| Intensive medical intervention | 28 (33.3) | 12 (42.8) | 16 (28.6) |
| Sleeve gastrectomy | 28 (33.3) | 8 (28.6) | 20 (35.7) |
| Roux-en-Y gastric bypass | 28 (33.3) | 8 (28.6) | 20 (35.7) |
| Fasting plasma glucose, mmol/L | 6.67±2.01 | 5.54±0.87 | 7.11±2.17^*^ |
| Hemoglobin A1c, % | 6.4±1.1 | 5.8±0.3 | 6.7±1.2^*^ |
| Total cholesterol, mg/dL | 184±39.5 | 188±31.0 | 181±42.8 |
| Triglyceride, mg/dL | 137±72.4 | 133±64.0 | 138±76.3 |
| Low-density lipoprotein cholesterol, mg/dL | 106±32.4 | 108±23.6 | 104±35.8 |
| High-density lipoprotein cholesterol, mg/dL | 50.9±11.5 | 54.3±13.5 | 49.3±10.2 |
| Fasting insulin, mU/L | 16.4±15.7 | 21.1±20.3 | 14.2±12.8 |
| Fasting proinsulin, pmol/L | 43.1±29.9 | 46.1±30.2 | 41.8±30.0 |
| Proinsulin/insulin ratio | 7.85±17.3 | 10.3±27.2 | 6.71±10.2 |
| Fasting C-peptide, pmol/L | 1082±486 | 1154±498 | 1049±480 |
| Unmethylated *INS* DNA, copies/μL | 1.44±1.46 | 0.97±0.44 | 1.66±1.70^*^ |
| Methylated *INS* DNA, copies/μL | 3.29±2.65 | 2.91±2.09 | 3.46±2.87 |
| Unmethylated/Methylated *INS* DNA ratio | 0.55±0.43 | 0.51±0.42 | 0.68±0.43^*^ |
| HOMA-IR | 0.26±0.25 | 0.29±0.29 | 0.24±0.23 |
| HOMA-β | 7.33±7.39 | 8.67±9.11 | 6.78±6.44 |

*INS* DNA, insulin gene DNA; HOMA-IR, homeostasis model assessment-estimated insulin resistance; HOMA-β, homeostatic Model Assessment of β-cell function.

All data were mean ± SD.

^*^Non-diabetes group vs. Type 2 diabetes group, *P* < 0.05.

Supplementary Figure 1. Flow chart of the study.


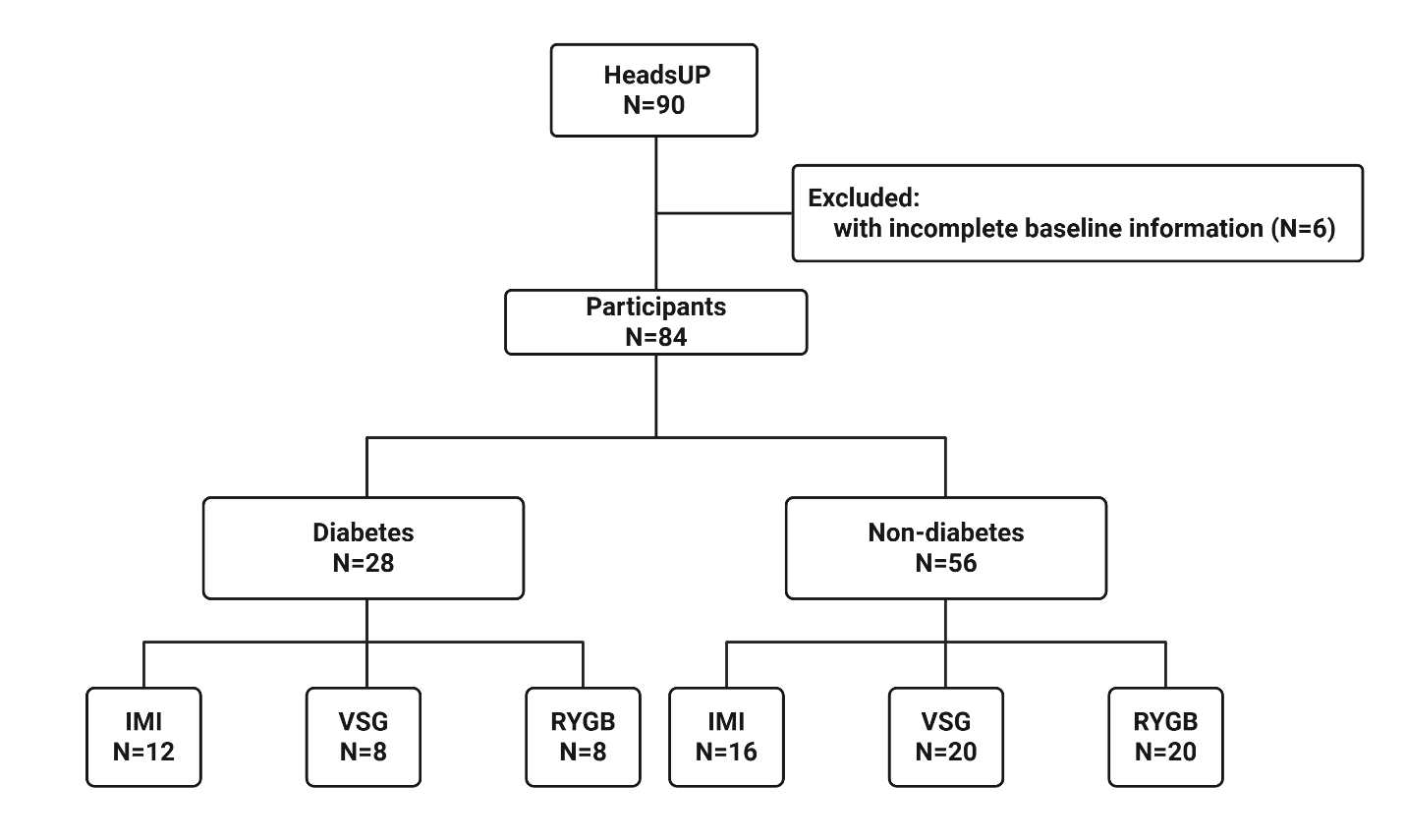


**
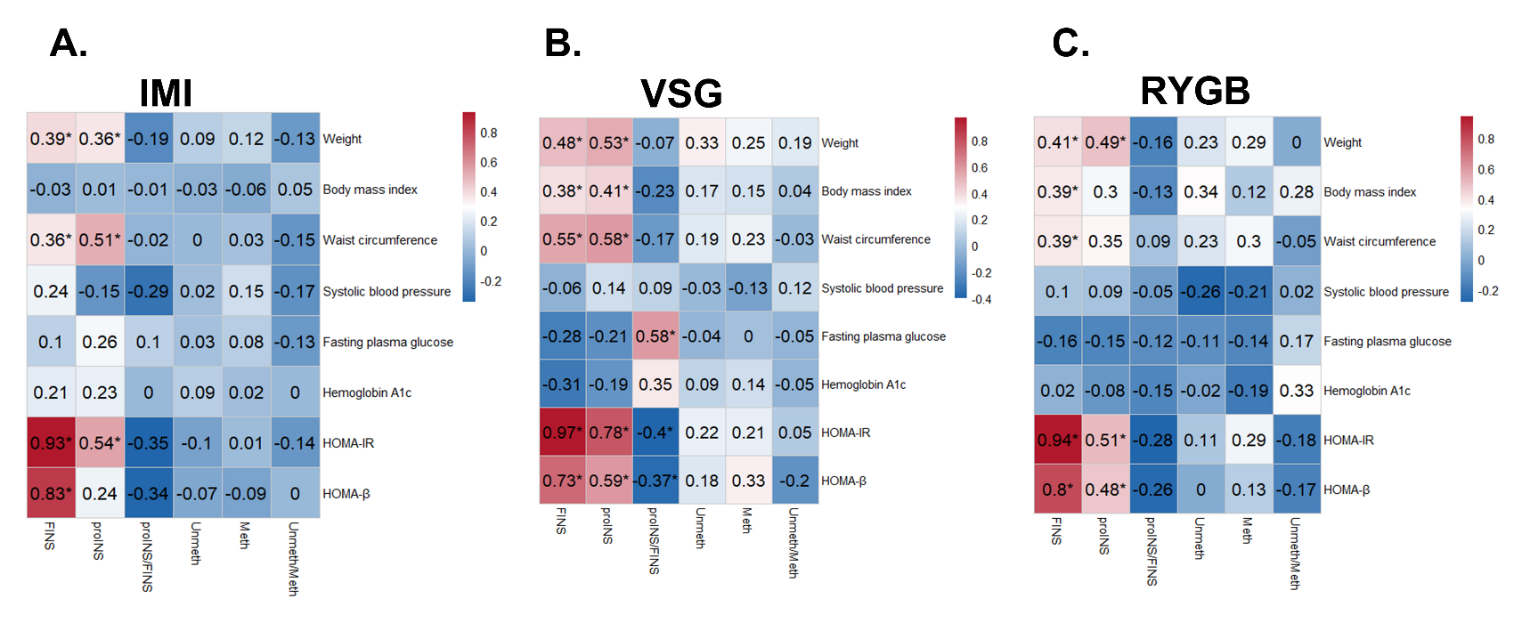
Supplementary Figure 2**. Correlation heatmaps of β cell traits with clinical characteristics among participants receiving different weight loss procedures. (A) Intensive medical intervention (IMI); (B) Vertical sleeve gastrectomy (VSG); (C) Roux-en-Y gastric bypass (RYGB). *, p < 0.05.
